# Capturing 3D large-strain Euler-bending filament dynamics in fibrous media simulations; sample case of compression collapse in dendritic actin network

**DOI:** 10.1038/s41598-019-40430-y

**Published:** 2019-03-08

**Authors:** Jyothirmai J. Simhadri, Preethi L. Chandran

**Affiliations:** Department of Chemical Engineering, Howard University, Washington, DC 2005 USA

## Abstract

Cytoskeletal networks to transmission towers are comprised of slender elements. Slender filaments bend and buckle more easily than stretch. Therefore a deforming network is expected to exhaust all possible bending-based modes before engaging filament stretch. While the large-strain bending critically determines fibrous-media response, simulations use small-strain and jointed approximations. At low resolution, these approximations inflate bending resistance and delay buckling onset. The proposed string-of-continuous-beams (SOCB) approach captures 3D nonlinear Euler bending of filaments with high fidelity at low cost. Bending geometry (i.e. angles and its differentials) is solved as primary variables, to fit a 5^th^ order polynomial of the contour angle. Displacement, solved simultaneously as length conservation, is predicted with C3 and C6 smoothness between and within segments, using only 2 nodes. In the chosen analysis frame, in-plane and out-plane moments can be decoupled for arbitrarily-curved segments. Complex crosslink force-transfers can be specified. Simulations show that when a daughter branch is appended, the buckling resistance of a filament changes from linear to nonlinear before reversible collapse. An actin outcrop with 8 generations of mother-daughter branching produced the linear, nonlinear, and collapse regimes observed in compression experiments. ‘Collapse’ was a redistribution of outcrop forces following the buckling of few strands.

## Introduction

From the actin network of the cytoskeleton to the cables of suspension bridges, nature and man have effectively used slender filaments for purposes of detection and structural support^1–4^. In a slender filament, the diameter is several times smaller than the length, producing two disparate mechanical resistances. One is the axial tensile resistance that is proportional to EA (E and A being elastic modulus and cross-sectional area, respectively), and the other is the bending resistance that is proportional to EI (I being the moment of inertia). Since A varies as the square of the diameter, I varies as the fourth power of the filament diameter, the bending resistance of a slender filament is several orders smaller than its tensile resistance. So while the tensile stiffness of slender filaments is used for load-bearing in structures (e.g. actin filaments in cells, cables in bridges), the bending compliance is used for amplifying strain in order to detect forces. For instance the stereocilia on ear cells^5^, cantilevers in an atomic force microscope^6^, and C-tube in Bourdon gauges^7^ are slender filaments that are used to detect micro-, kilo-, and mega- pascals of pressure, respectively. The filament deflection amplifies the pressure signal for a sensor in series (e.g. ion channels, photo detectors, and capacitors), which transduces the pressure signal to charge currents.

Soft slender media such as tissue and cell matrices have evolved to synergistically utilize the tensile load-bearing and bending strain-amplification properties of filaments. In cell and tissue matrices, bending deformations allow an arrangement of stiff filaments to be compliant at low tensile and compressive strains^8–14^. The buckling of filaments in compression allows cellular networks to generate strong-directional anisotropies in stress resistance^15^, which transmit long distances un-attenuated, such as in the case of stress fibers^16,17^ and matrix tethers^18,19^. The sites of high curvature in buckled actin filaments serve as sites for enzymatic cleavage, imparting local fluidity to a rearranging cytoskeletal network^20,21^.

Since bending is an energetically cheaper mode, a filamentous matrix can be expected to extinguish all possible strain-transmission routes that involve large-strain filament bending before recruiting the filaments to stretch^8–10^. Moreover bending filaments can be tipped into buckling, where large displacements occur without much cost to the strain energy, giving filamentous media ample flexibility for routing internal strains using filament bending modes alone. Therefore one can argue that capturing large-strain geometries and mechanics of filament bending is important in simulations of filamentous networks^1,22^. While nonlinearities in filament axial mechanics can be measured and simulated with high fidelity, the bending geometry and mechanics of filaments is usually approximated with linear or jointed spring assumptions in large-scale filamentous networks. In cases where the nonlinear bending geometry is captured with high fidelity, the mathematical formulations are tailored for specific filament configurations and boundary conditions, and in many cases 2D space^23–27^. Generalized solutions are needed to apply for the range of 3D contours, arrangements, loading, and boundary conditions a filament can assume within realistic filamentous media that can also be dynamically changing (such as the myosin- and cell- remodeling of cytoskeletal and tissue networks, respectively)^28–32^.

In this paper we present a universal approach to solving nonlinear shear-free bending in arbitrary filamentous media in 3D space. The strategy is based on two ideas: The first is to take advantage of the linear proportionality or coupling between the mechanics and geometry terms in Euler bending equations by retaining the geometric terms as primary variables instead of approximating them to displacement variables (Sec. A under Theory). By doing so, the angle contour can be determined to fifth order continuity using only the mechanics variables at two nodes. The displacements are instead determined from mass-conservation (or length-conservation for a filament) equations solved simultaneously with the mechanics equations. The second idea is to generalize the solution procedure for arbitrary filament contours in 3D by performing the moment balances in a rotated frame where the filament shadow on each axis becomes the moment arm for the bending forces (Sec. B under Theory). Compared to conventional modeling approaches for arbitrary filament meshes, the proposed method captures nonlinear bending forces and conformations with high accuracy at reduced computational cost. Since the geometry terms are solved as primary variables, complex crosslink physics can be applied at filament interconnections. For instance, one can specify the stress-continuous but materially-discontinuous nature of filament interconnections in dendritic actin meshes where daughter filaments offshoot from proteins bound to the mother filament. The compression of a dendritic actin outcrop with eight tiers of offshoots was simulated with regular computational resources. The simulations reproduced the signature features of toe region, nonlinear stiffening, and reversible collapse documented by Chaudhari *et al*.^33^.

## Theory

We present the theoretical aspects of the String of Continuous Beams (SOCB) approach that enable the solution of large-strain bending in arbitrary slender media. Details on how the theory is implemented, with step-by-step descriptions of the algorithm, can be found in Supplementry Information 1.

### Coupling the bending mechanics and geometry

The difficulty in capturing the nonlinear bending geometry of a slender filament can be regarded as a problem of inefficiently enforcing the coupling between mechanics and nonlinear geometry prescribed in large-strain Euler bending. Euler beams (as opposed to Timoshenko beams) assume shear-free bending; i.e. the filament cross section is always perpendicular to the bending line (line of zero extension or compression), which is generally regarded valid for bending in slender elements where aspect ratio is greater than 10^34^. In the large-strain Euler bending equations, the mechanical variables (i.e. bending force *F* and moment *M*) are directly proportional to the geometric variables (i.e. curvature ${\rm{d}}{\rm{\theta }}/{\rm{d}}s(s)$ and its differential ${{\rm{d}}}^{2}\theta /{{\rm{d}}s}^{2}(s)$):

1 $${\rm{M}}({\rm{s}})=-\,{\rm{EI}}\frac{{\rm{d}}{\rm{\theta }}}{{\rm{ds}}}({\rm{s}})$$

2 $${\rm{F}}({\rm{s}})={\rm{EI}}\frac{{{\rm{d}}}^{2}{\rm{\theta }}}{{{\rm{ds}}}^{2}}({\rm{s}})$$

Here $\theta (s)$ is the in-plane or 2D bending angle at distance *s* along the contour of a filament. However, instead of using the mechanics variables to directly predict the bending contour of a filament, most modeling approaches apply small-strain (Eq. 3) and ‘jointed’ (Eq. 4) approximations to make the calculation of geometric variables like curvature tenable for rectangular coordinates.

3 $${\rm{Small}}\,{\rm{strain}}\,{\rm{approximation}}:{\rm{d}}{\rm{\theta }}/{\rm{ds}}=\frac{{{\rm{d}}}^{2}{\rm{y}}/{{\rm{dx}}}^{2}}{{[1+{({\rm{dy}}/{\rm{dx}})}^{2}]}^{3/2}}\approx {{\rm{d}}}^{2}{\rm{y}}/{{\rm{dx}}}^{2}$$

4 $${\rm{Jointed}}\,{\rm{approximation}}:{\rm{d}}{\rm{\theta }}/{\rm{ds}}={{\rm{d}}}^{2}{\rm{y}}/{{\rm{dx}}}^{2}\approx \frac{1}{{\rm{L}}}{\rm{\Delta }}[{\rm{dy}}/{\rm{dx}}]=\frac{1}{{\rm{L}}}{\rm{\Delta }}[{\rm{\theta }}]$$

**Figure 1:**
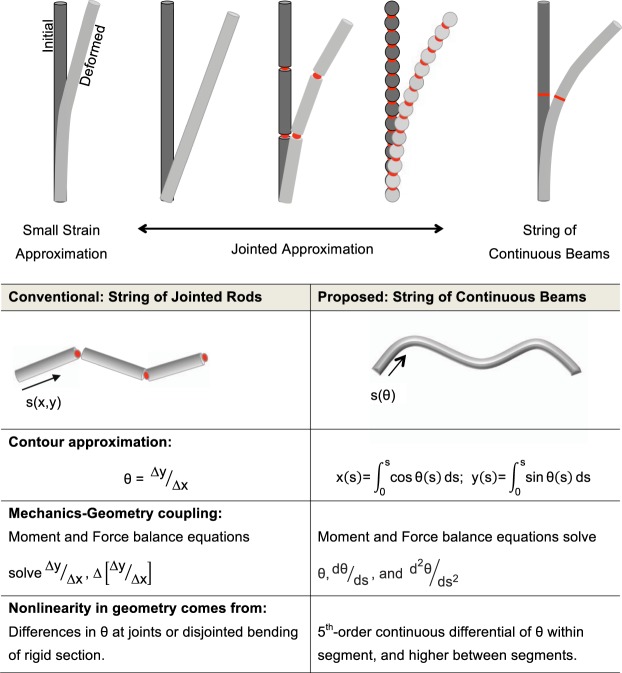
(Top) The bending deformation of a slender filament in different modeling idealizations. The proposed SOCB idealization does not rely on the small-strain or jointed approximation. (Bottom) Listing of the principal differences between the oft-used jointed and proposed SOCB approached, presented for the sample case of 2D bending.

These approximations break down the direct proportionality between mechanics and nonlinear geometry naturally afforded by the Euler force and moment equations^23^. The small-strain approximation is known to introduce an artifactual lengthening of the bending filament (Fig. 1, top panel)^34^. In the ‘jointed’ approximation, the continuous resistance to angle change present in a bending filament is restricted to occurring only at hinge joints connecting straight and rigid segments of filaments (Fig. 1, top panel)^35,36^. The jointed or discontinuous-filament approximation is common for modeling slender elements from the structural engineering to the polymer field, with lower discretization more prevalent in the former and higher discretization (tending towards string-of-beads approach) more common in the latter (Fig. 1, top panel). However, the systematic error introduced by the jointed approximation is not clear.

In order to preserve the proportionality between filament mechanics and nonlinear geometry, we propose a simple change to how nonlinear bending mechanics is solved in filament models (Fig. 1, bottom panel). We propose the string-of-continuous-beams (SOCB) approach where the contour angle and its differentials in the beam mechanics equations (Eqs 1 and 2) are retained as primary variables for those equations instead of approximating to displacements^9,10,37,38^.

**Figure 2:**
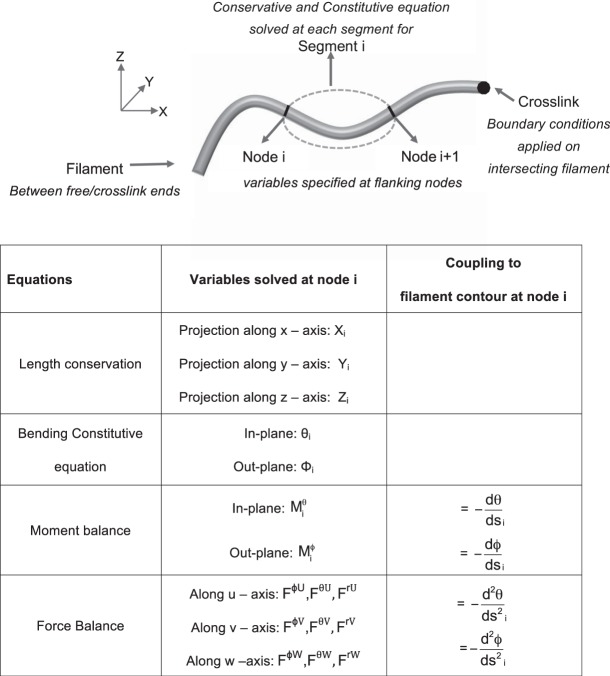
(**A**) Each stretch of slender element between crosslinks or free ends is considered a filament. A stretch of filament between two nodes is a segment. The conservation (length, force, moment) and constitutive equations are solved for each segment. (**B**) The table lists the equations assembled at each segment, the principle variables of beam mechanics being solved at each node, and the counterpart geometric variables ‘coupled’ to filament conformation. The latter are not reduced to be expressed in terms of displacements, but rather contribute to the polynomial interpolating the angle change between the two nodes.

This approach enhances the geometric information obtained from solving only two nodes of a filament. For instance, consider a filament segment bending only in the plane of the latitudes in spherical coordinates (i.e. only the in-plane bending angle $\theta (s)$ changes - Fig. 2). The segment is flanked by nodes i and i + 1 at its two ends. The primary variables determined upon solving the force, moment, and bending equations on the segment are: ${{\rm{\theta }}}_{{\rm{i}}}\,{\rm{and}}\,{{\rm{\theta }}}_{{\rm{i}}+1},\,{\rm{d}}{\rm{\theta }}/{{\rm{ds}}}_{{\rm{i}}}\,{\rm{and}}\,{\rm{d}}{\rm{\theta }}/{{\rm{ds}}}_{{\rm{i}}+1},\,{{\rm{d}}}^{2}{\rm{\theta }}/{{\rm{ds}}}_{{\rm{i}}}^{2}\,{\rm{and}}\,{{\rm{d}}}^{2}{\rm{\theta }}/{{\rm{ds}}}_{{\rm{i}}+1}^{2}$. Instead of reducing these variables to be expressed in terms of displacement, we use them to determine the coefficients of a fifth-order polynomial describing how ${\rm{\theta }}({\rm{s}})\,\,$changes between the two nodes:

5 $${\rm{\theta }}({\rm{s}})={{\rm{a}}}^{{\rm{\theta }}}+{{\rm{b}}}^{{\rm{\theta }}}{{\rm{s}}}^{1}+{{\rm{c}}}^{{\rm{\theta }}}{{\rm{s}}}^{2}+{{\rm{d}}}^{{\rm{\theta }}}{{\rm{s}}}^{3}+{{\rm{e}}}^{{\rm{\theta }}}{{\rm{s}}}^{4}+{{\rm{f}}}^{{\rm{\theta }}}{{\rm{s}}}^{5}$$

The coefficients are determined by substituting s = 0 for values at node i, and s = l for values at node i + 1 in correspondingly differentiated forms of Eq. 5; l is the segment length. The resulting set of simultaneous equations (Eq. 6a) includes a ‘coupling matrix’ that ties the mechanical variables to the contour geometry via the coefficients of the angle-evolution polynomial in Eq. 5.

6a $$[\begin{array}{c}\begin{array}{c}\begin{array}{c}{\theta }_{{\rm{i}}}\\ {{\rm{M}}}_{{\rm{i}}}\\ {{\rm{V}}}_{{\rm{i}}}^{\theta }\end{array}\\ {\theta }_{{\rm{i}}+1}\\ {{\rm{M}}}_{{\rm{i}}+1}\\ {{\rm{V}}}_{{\rm{i}}+1}^{\theta }\end{array}\end{array}]=[\begin{array}{c}\begin{array}{c}\begin{array}{c}{\theta }_{{\rm{i}}}\\ \frac{{\rm{d}}\theta }{{{\rm{d}}{\rm{s}}}_{{\rm{i}}}}\\ \frac{{{\rm{d}}}^{2}\theta }{{{\rm{d}}{\rm{s}}}_{{\rm{i}}}^{2}}\end{array}\\ {\theta }_{{\rm{i}}+1}\\ \frac{{\rm{d}}\theta }{{{\rm{d}}{\rm{s}}}_{{\rm{i}}+!}}\\ \frac{{{\rm{d}}}^{2}\theta }{{{\rm{d}}{\rm{s}}}_{{\rm{i}}+!}^{2}}\end{array}\end{array}]=[\begin{array}{c}\begin{array}{cccccc}1 & 0 & 0 & 0 & 0 & 0\\ 0 & 1 & 0 & 0 & 0 & 0\\ 0 & 0 & 1 & 0 & 0 & 0\\ 1 & {\rm{l}} & {{\rm{l}}}^{2} & {{\rm{l}}}^{3} & {{\rm{l}}}^{4} & {{\rm{l}}}^{5}\\ 0 & 1 & 2{\rm{l}} & 3{{\rm{l}}}^{2} & 4{{\rm{l}}}^{3} & 5{{\rm{l}}}^{4}\\ 0 & 0 & 2 & 6{\rm{l}} & {12{\rm{l}}}^{2} & {20{\rm{l}}}^{3}\end{array}\end{array}]\,[\begin{array}{c}\begin{array}{c}\begin{array}{c}{{\rm{a}}}^{\theta }\\ {{\rm{b}}}^{\theta }\\ {{\rm{c}}}^{\theta }\end{array}\\ {{\rm{d}}}^{\theta }\\ {{\rm{e}}}^{\theta }\\ {{\rm{f}}}^{\theta }\end{array}\end{array}]$$

A similar ‘coupling matrix’ equation is written for the out-of-plane angle. The x-, y-, z- displacement variables simultaneously solved from the angle-evolution polynomials using standard spherical coordinate projections as part of the mass-conservation equations.

SOCB length conservation equations:

7 $$\begin{array}{rcl}{{\rm{X}}}_{{\rm{i}}+{\rm{1}}}-{{\rm{X}}}_{{\rm{i}}} & = & {\int }_{0}^{l}\,\cos \,{\rm{\theta }}({\rm{s}})\cos \,{\rm{\varphi }}({\rm{s}}){\rm{ds}}\\ {{\rm{Y}}}_{{\rm{i}}+{\rm{1}}}-{{\rm{Y}}}_{{\rm{i}}} & = & {\int }_{0}^{l}\,\sin \,{\rm{\theta }}({\rm{s}})\cos \,{\rm{\varphi }}({\rm{s}}){\rm{ds}}\\ {Z}_{{\rm{i}}+{\rm{1}}}-{{\rm{Z}}}_{{\rm{i}}} & = & {\int }_{0}^{l}\,\sin \,{\rm{\varphi }}({\rm{s}}){\rm{ds}}\end{array}$$

The displacements are determined by numerically-integrating the angle polynomial in a length conservation equation solved simultaneously with the mechanics. In other  words, the mechanics/geometry variables intrinsically satisfy segment inextensibility and displacement continuity. Figure 2 describes the coupling between mechanics and geometric terms in the 3D bending equations. 5^th^ order continuity in angle is obtained by efficiently integrating the geometric information across the force, momentum, and constitutive equations solved at only two nodes. This approach is much different than that of Finite Element analysis, where high-order interpolation is achieved by increasing the nodes where variables are solved within a segment or element (p-refinement). Also, the SOCB approach reduces to the jointed or hinged approach (C0 continuous between segments) when all coefficients but *a* are set to zero in Eqs 5 and 6. (Supplementry Information 2). Moreover, the nodes are placed at the ends and intersections of continuous segments in the SOCB approach (Fig. 2). Therefore the two segment ends meeting at the node have the same values for displacements, angles, curvature, and curvature differential. In other words, displacements and angles are respectively C3 and C2 continuous between segments in the SOCB approach.

### Generalizing for arbitrary filament arrangements in 3D

The bending solution described above needs to be generalized for arbitrary filament conformations and loading in 3D. A challenge for such generalizations is calculating the moment arms of 3D forces on the arbitrary 3D filament contours^39,40^. In the SOCB approach, an analysis frame is chosen where the filament projections along the frame axis also become the moment arms for the bending force components.

**Figure 3:**
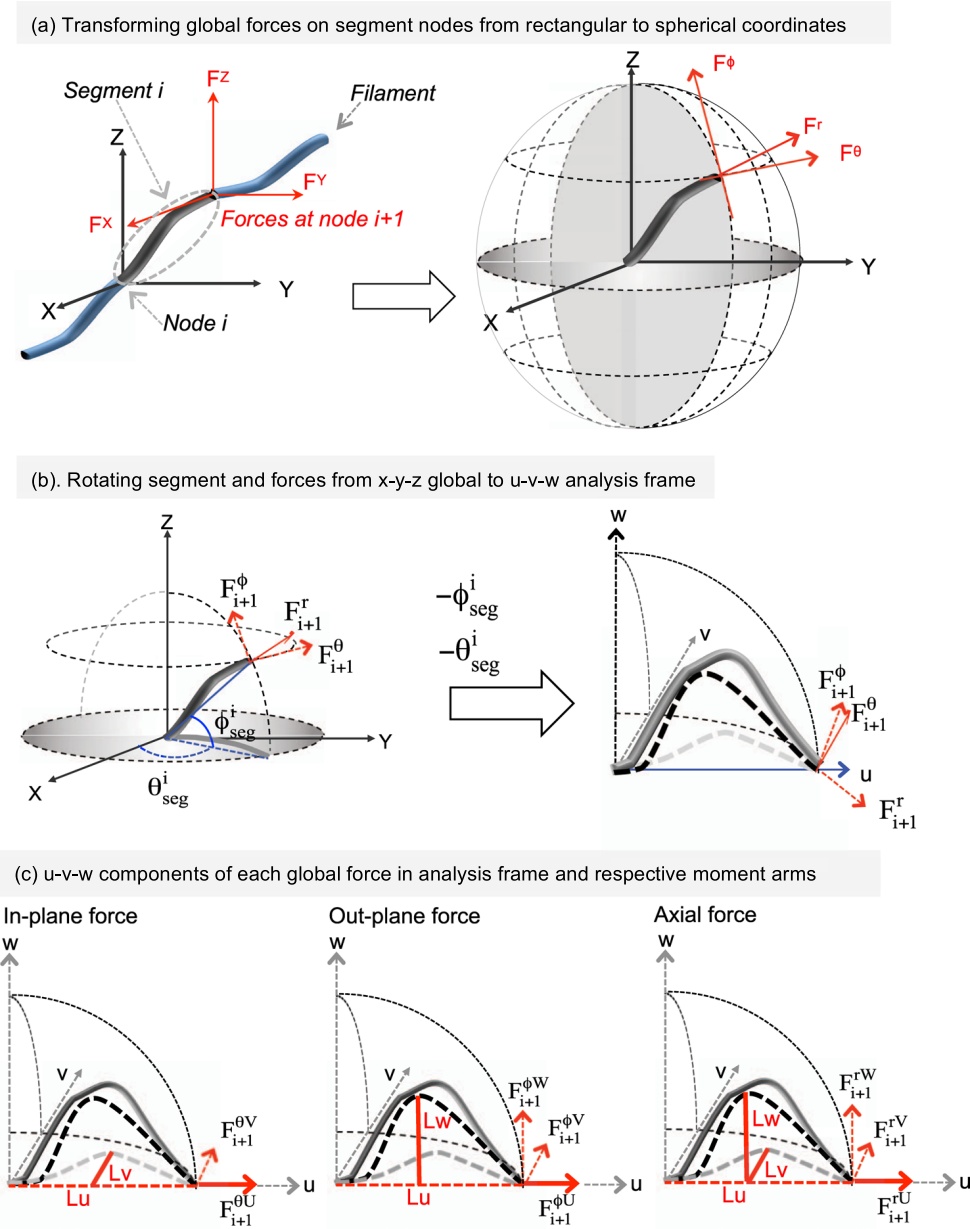
Determining bending forces and moment arms for an arbitrary curved segment: (**a**) The forces at the nodes (i.e. ends of a segment) of a curved filament in global coordinates are decomposed into the three mutually-perpendicular directions: the radial r (tangential to filament), in-plane θ (tangential to latitude), and out-plane (tangential to longitude) directions at node i + 1 are shown. The in-plane and out-plane forces causes out-of-axis bending in the latitudinal (X–Y) and longitudinal plane, respectively. The radial force on a curved filament causes (un)folding in the plane containing the radial force and its moment arm. (**b**) To determine the moment arms for the global forces on node i + 1, the segment is rotated into an analysis u-v-w frame, which is aligned along the global x-y-z frame. ϕ_seg_ and θ_seg_ are the out- and in-plane angles of the line joining the end nodes of a segment i, and rotation through these angles aligns the segment line with the x axis of the global frame or u-axis of the analysis frame. The rotation occurs along the lines of latitude and longitude, and does not change the in-plane, out-plane, and axial character of the forces on node i + 1. The dark and light dotted contours represent the filament shadow in the u-w (longitudinal) and u-v (latitudinal) planes, respectively. (**c**) The moment arms assignment is shown separately for the three forces, and in each figure the line pattern of the force component and its moment are kept similar. The in-plane, out-plane, and radial forces of node i + 1 are decomposed into their respective u, v, and w components. Their respective moment arm (Lu, Lv, or Lw) should be perpendicular to the force component and lie in plane of bending.

Consider an arbitrarily curved segment i between two nodes i and i + 1 in 3D space (Fig. 3a). The global x-, y-, and z- forces at a node can be decomposed into in-plane, out-plane and axial directions at the node (Fig. 3a). The in-plane and out-plane forces act tangential to the lines of latitude and longitude, respectively, passing through a node, and cause bending along those planes. Only the in-plane forces contribute to the in-plane bending moment equation, and only the out-plane forces contribute to the out-plane bending moment equation. However, the axial force at node i + 1 can contribute to bending (i.e. folding or unfolding) the segment in either plane, if the contour has an inflexion and projects in the respective bending plane (Fig. 3a, right).

The arbitrary-curved segment in the global frame (x-y-z) is rotated into the analysis frame (u-v-w) through spherical angles ϕ and θ of the line joining its two ends, i.e. through ${{\rm{\varphi }}}_{{\rm{seg}}}^{{\rm{i}}}\,{\rm{and}}\,{{\rm{\theta }}}_{{\rm{seg}}}^{{\rm{i}}}$ (Fig. 3b). Note the u, v, w axes are aligned along the original x, y, z ones. The rotation lines up the two segment nodes along ‘u’ of the u, v, w analysis frame (or x in the global frame). Since the rotation occurs along the latitudes and longitudes, it does not change the in-plane and out-plane character of the nodal forces on the segment. For instance, the u-v plane will now have the in-plane nodal forces and bending (Fig. 3b).

Each of the global forces on node i + 1 are decomposed into their respective u, v, and w components. While the out-plane (bending along longitude) and radial forces have components in the u, v, and w directions, the in-plane (bending along latitude) force has components only in the u and v directions (Fig. 3c). The segment projections along the u, v, and w directions (i.e. Lu, Lv, and Lw, respectively) directly provide the moment arms for the u, v, and w components of each of the global forces ${{\rm{F}}}_{{\rm{i}}+1}^{{\rm{\varphi }}},\,{{\rm{F}}}_{{\rm{i}}+1}^{{\rm{\theta }}}\,{\rm{and}}\,{{\rm{F}}}_{{\rm{i}}+1}^{{\rm{r}}}$ on the node i + 1. Now a moment arm should be perpendicular to the force component and lie on the plane of bending (i.e. perpendicular to the bending axis) assigned for the force component. Therefore the moment arm for the w component of ${{\rm{F}}}_{{\rm{i}}+1}^{{\rm{\varphi }}}$ should be perpendicular to w direction and lie on the u-w out-plane. It would therefore be Lu, the filament projection along the u axis. Lu will also be the moment arm for the v component of ${{\rm{F}}}_{{\rm{i}}+1}^{{\rm{\varphi }}}$ which causes bending in the in-plane (u-v). Similarly Lv will be the moment arm for the u component of the in-plane force ${{\rm{F}}}_{{\rm{i}}+1}^{{\rm{\varphi }}}$. In Fig. 3c, lines of similar thickness and pattern are used to distinguish a force component and it corresponding moment arm. Note the global radial force will cause the segment to fold or unfold, in the plane in which it has a projected length or moment arm.

Since the in- and out- bending character of the forces were not changed with rotation into the analysis frame, the bending moments calculated in the analysis frame can be directly transmitted to the global moment balance. Table 1 lists the torque terms that contribute to the in-plane and out-plane moment conservation equations. All moment arms for the in-plane moment balance, calculated for the u-v plane, have to be scaled by $\cos ({{\rm{\varphi }}}_{{\rm{seg}}}^{{\rm{i}}})\,\,$so that they match with the in-plane moment arms in the global frame. This is because when a filament rotates into the u-v-w analysis frame, its latitudinal plane in which in-plane bending occurs will expand. A pictorial depiction is shown in Suppl. Info 3. To correct for this expansion, all projections or moment-arms contributing to the calculation of in-plane bending in the analysis frame are compressed by a factor of $\cos ({{\rm{\varphi }}}_{{\rm{seg}}}^{{\rm{i}}})$ (Table 1). The compression of in-plane moment-arms with $\cos ({{\rm{\varphi }}}_{{\rm{seg}}}^{{\rm{i}}})$ addresses the asymmetry between the in-plane and out-plane bending displacements in spherical coordinates, and removes the singularity that occurs when the segment is pointed in the z direction in spherical coordinates (i.e. $\cos ({{\rm{\varphi }}}_{{\rm{seg}}}^{{\rm{i}}})=0$ when ${\varphi }_{{\rm{s}}{\rm{e}}{\rm{g}}}^{{\rm{i}}}=90$). The moment conservation equations (Eq. 8) for the in-plane and out-plane bending can be written from the torques listed in Table 1.

**Table 1:** Contribution to the force and moment balance equations (rows) from each of the in-plane, out-plane, and radial global forces (columns). The latitudinal moment arms are compressed by $\cos ({{\rm{\varphi }}}_{{\rm{seg}}}^{{\rm{i}}})$ to correct for the expansion in moment arm that occurs when a segment is rotated into the analysis frame.

In-plane moment balance	$\cos ({\varphi }_{{\rm{s}}{\rm{e}}{\rm{g}}}^{{\rm{i}}})[{{\rm{F}}}_{{\rm{i}}+1}^{\theta {\rm{V}}}{{\rm{L}}}_{{\rm{U}}}-{{\rm{F}}}_{{\rm{i}}+1}^{\theta {\rm{U}}}{{\rm{L}}}_{{\rm{V}}}]$		$\cos ({\varphi }_{{\rm{s}}{\rm{e}}{\rm{g}}}^{{\rm{i}}})[{{\rm{F}}}_{{\rm{i}}+1}^{{\rm{r}}{\rm{V}}}{{\rm{L}}}_{{\rm{U}}}-{{\rm{F}}}_{{\rm{i}}+1}^{{\rm{r}}{\rm{U}}}{{\rm{L}}}_{{\rm{V}}}]$
Out-plane moment balance		${{\rm{F}}}_{{\rm{i}}+{\rm{1}}}^{{\rm{\varphi }}{\rm{W}}}{{\rm{L}}}_{{\rm{U}}}-{{\rm{F}}}_{{\rm{i}}+{\rm{1}}}^{{\rm{\varphi }}{\rm{U}}}{{\rm{L}}}_{{\rm{W}}}-{{\rm{F}}}_{{\rm{i}}+{\rm{1}}}^{{\rm{\varphi }}{\rm{V}}}{{\rm{L}}}_{{\rm{W}}}$	${{\rm{F}}}_{{\rm{i}}+{\rm{1}}}^{{\rm{rW}}}{{\rm{L}}}_{{\rm{U}}}-{{\rm{F}}}_{{\rm{i}}+{\rm{1}}}^{{\rm{rU}}}{{\rm{L}}}_{{\rm{W}}}$
u - Force balance	${{\rm{F}}}_{{\rm{i}}+{\rm{1}}}^{{\rm{\theta }}{\rm{U}}}$	${{\rm{F}}}_{{\rm{i}}+{\rm{1}}}^{{\rm{\varphi }}{\rm{U}}}$	${{\rm{F}}}_{{\rm{i}}+{\rm{1}}}^{{\rm{rU}}}$
v - Force balance	${{\rm{F}}}_{{\rm{i}}+{\rm{1}}}^{{\rm{\theta }}{\rm{V}}}$	${{\rm{F}}}_{{\rm{i}}+{\rm{1}}}^{{\rm{\varphi }}{\rm{V}}}$	${{\rm{F}}}_{{\rm{i}}+{\rm{1}}}^{{\rm{\varphi }}{\rm{V}}}$
w - Force balance		${{\rm{F}}}_{{\rm{i}}+{\rm{1}}}^{{\rm{\varphi }}{\rm{W}}}$	${{\rm{F}}}_{{\rm{i}}+{\rm{1}}}^{{\rm{\varphi }}{\rm{W}}}$

SOCB moment conservation equations:

8a $${{\rm{M}}}_{{\rm{i}}+1}^{\theta }-{{\rm{M}}}_{{\rm{i}}}^{\theta }=\,\cos ({\varphi }_{{\rm{s}}{\rm{e}}{\rm{g}}}^{{\rm{i}}})[{{\rm{F}}}_{{\rm{i}}+1}^{\theta {\rm{V}}}{{\rm{L}}}_{{\rm{U}}}-{{\rm{F}}}_{{\rm{i}}+1}^{\theta {\rm{U}}}{{\rm{L}}}_{{\rm{V}}}]+\,\cos ({\varphi }_{{\rm{s}}{\rm{e}}{\rm{g}}}^{{\rm{i}}})[{{\rm{F}}}_{{\rm{i}}+1}^{{\rm{r}}{\rm{V}}}{{\rm{L}}}_{{\rm{U}}}-{{\rm{F}}}_{{\rm{i}}+1}^{{\rm{r}}{\rm{U}}}{{\rm{L}}}_{{\rm{V}}}]$$

8b $${{\rm{M}}}_{{\rm{i}}+{\rm{1}}}^{{\rm{\varphi }}}-{{\rm{M}}}_{{\rm{i}}}^{{\rm{\varphi }}}={{\rm{F}}}_{{\rm{i}}+{\rm{1}}}^{{\rm{\varphi }}{\rm{W}}}{{\rm{L}}}_{{\rm{U}}}-{{\rm{F}}}_{{\rm{i}}+{\rm{1}}}^{{\rm{\varphi }}{\rm{U}}}{{\rm{L}}}_{{\rm{W}}}-{{\rm{F}}}_{{\rm{i}}+{\rm{1}}}^{{\rm{\varphi }}{\rm{V}}}{{\rm{L}}}_{{\rm{W}}}+{{\rm{F}}}_{{\rm{i}}+{\rm{1}}}^{{\rm{rW}}}{{\rm{L}}}_{{\rm{U}}}-{{\rm{F}}}_{{\rm{i}}+{\rm{1}}}^{{\rm{rU}}}{{\rm{L}}}_{{\rm{W}}}$$

where ${{\rm{M}}}_{{\rm{i}}}^{{\rm{\theta }}}$ and ${{\rm{M}}}_{{\rm{i}}}^{{\rm{\varphi }}}$ are the in-plane and out-plane bending moments on node i. $\cos ({\varphi }_{{\rm{s}}{\rm{e}}{\rm{g}}}^{{\rm{i}}})$ is the out-plane angle of the straight line connecting the ends of segment i. ${{\rm{F}}}_{{\rm{i}}+1}^{{\rm{\theta }}{\rm{V}}},\,{{\rm{F}}}_{{\rm{i}}+1}^{{\rm{\varphi }}{\rm{V}}}$and ${{\rm{F}}}_{{\rm{i}}+1}^{{\rm{rV}}}$ are the v-components of the in-plane, out-plane, and radial forces on node i + 1, respectively, in the rotated frame. u- and w- projections are labeled similarly.

The above moment conservation equations specify how the curvatures $(\frac{{\rm{d}}{\rm{\theta }}}{{\rm{ds}}},\frac{{\rm{d}}{\rm{\varphi }}}{{\rm{ds}}})$ change between the two segment ends. The change in angle between the two ends can be obtained using the bending constitutive equation. In methods where the bending mechanics and curvature is not coupled, a constitutive equation such as Eq. 4 needs to be specified to relate the bending moment M to the difference in angles. However, in a fully coupled scheme such as the SOCB method, ${{\rm{M}}}^{{\rm{\theta }}}\infty \frac{{\rm{d}}{\rm{\theta }}}{{\rm{ds}}}$ and therefore ${\rm{d}}{\rm{\theta }}\infty {\int }_{{\rm{o}}}^{{\rm{l}}}{{\rm{M}}}^{{\rm{\theta }}}{\rm{ds}}$, and so forth for the out-plane. The bending constitutive equation, which determines the difference in nodal angles, becomes simply the integral of the difference between the nodal moments or the integral of the moment conservation equation (Eq. 9).

SOCB bending constitutive equation for angle change:

9a $${\theta }_{{\rm{i}}+1}-{\theta }_{{\rm{i}}}={[\cos ({\varphi }_{{\rm{s}}{\rm{e}}{\rm{g}}}^{{\rm{i}}})]}^{2}[{{\rm{F}}}_{{\rm{i}}+1}^{\theta {\rm{V}}}\frac{{[{{\rm{L}}}_{{\rm{U}}}]}^{2}}{2}-{{\rm{F}}}_{{\rm{i}}+1}^{\theta {\rm{U}}}\frac{{[{{\rm{L}}}_{{\rm{V}}}]}^{2}}{2}]+{[\cos ({\varphi }_{{\rm{s}}{\rm{e}}{\rm{g}}}^{{\rm{i}}})]}^{2}[{{\rm{F}}}_{{\rm{i}}+1}^{{\rm{r}}{\rm{V}}}\frac{{[{{\rm{L}}}_{{\rm{U}}}]}^{2}}{2}-{{\rm{F}}}_{{\rm{i}}+1}^{{\rm{r}}{\rm{U}}}\frac{{[{{\rm{L}}}_{{\rm{V}}}]}^{2}}{2}]$$

9b $${\varphi }_{{\rm{i}}+1}-{\varphi }_{{\rm{i}}}={{\rm{F}}}_{{\rm{i}}+1}^{\varphi {\rm{W}}}\frac{{[{{\rm{L}}}_{{\rm{U}}}]}^{2}}{2}-{{\rm{F}}}_{{\rm{i}}+1}^{\varphi {\rm{U}}}\frac{{[{{\rm{L}}}_{{\rm{W}}}]}^{2}}{2}-{{\rm{F}}}_{{\rm{i}}+1}^{\varphi {\rm{V}}}\frac{{[{{\rm{L}}}_{{\rm{W}}}]}^{2}}{2}+{{\rm{F}}}_{{\rm{i}}+1}^{{\rm{r}}{\rm{W}}}\frac{{[{{\rm{L}}}_{{\rm{U}}}]}^{2}}{2}-{{\rm{F}}}_{{\rm{i}}+1}^{{\rm{r}}{\rm{U}}}\frac{{[{{\rm{L}}}_{{\rm{W}}}]}^{2}}{2}$$

Finally, the momentum conservation equation for the arbitrarily-curve segment becomes a static balance of force components in the u, v, and w directions in the analysis frame (Eq. 10).

SOCB force conservation equations:

10a $${{\rm{F}}}_{{\rm{i}}+1}^{{\rm{\theta }}{\rm{U}}}+{{\rm{F}}}_{{\rm{i}}+1}^{{\rm{\varphi }}{\rm{U}}}+{{\rm{F}}}_{{\rm{i}}+1}^{{\rm{rU}}}-{{\rm{F}}}_{{\rm{i}}}^{{\rm{\theta }}{\rm{U}}}-{{\rm{F}}}_{{\rm{i}}}^{{\rm{\varphi }}{\rm{U}}}-{{\rm{F}}}_{{\rm{i}}}^{{\rm{rU}}}=0$$

10b $${{\rm{F}}}_{{\rm{i}}+1}^{\theta {\rm{V}}}+{{\rm{F}}}_{{\rm{i}}+1}^{\varphi {\rm{V}}}+{{\rm{F}}}_{{\rm{i}}+1}^{{\rm{r}}{\rm{V}}}-{{\rm{F}}}_{{\rm{i}}}^{\theta {\rm{V}}}-{{\rm{F}}}_{{\rm{i}}}^{\varphi {\rm{V}}}-{{\rm{F}}}_{{\rm{i}}}^{{\rm{r}}{\rm{V}}}=0$$

10c $${{\rm{F}}}_{{\rm{i}}+1}^{\theta {\rm{W}}}+{{\rm{F}}}_{{\rm{i}}+1}^{\varphi {\rm{W}}}+{{\rm{F}}}_{{\rm{i}}+1}^{{\rm{r}}{\rm{W}}}-{{\rm{F}}}_{{\rm{i}}}^{\theta {\rm{W}}}-{{\rm{F}}}_{{\rm{i}}}^{\varphi {\rm{W}}}-{{\rm{F}}}_{{\rm{i}}}^{{\rm{r}}{\rm{W}}}=0$$

## Results

### Validation of the mechanics-geometry coupling approach

**Figure 4:**
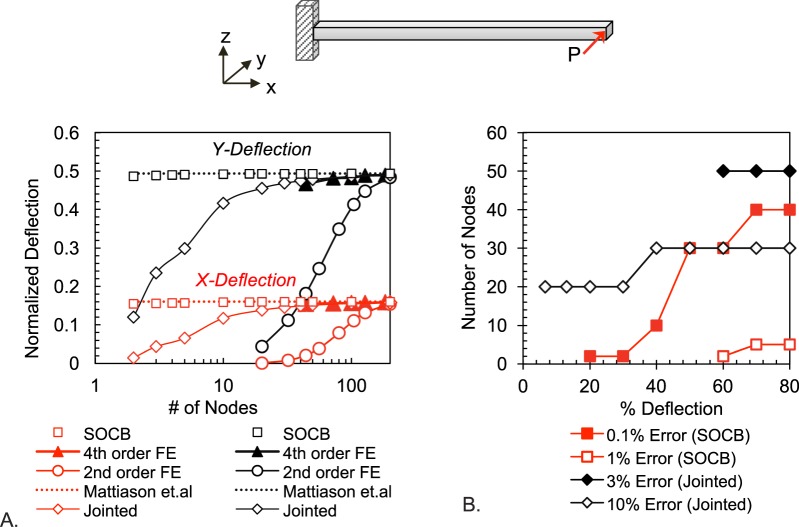
(**A**) Error in predicting Y- and X- off axis cantilever deflection for increasing node numbers in the proposed SOCB model and in the gold-standard FEA and jointed-model approaches: An end load P scaled so that PL^2^/EI = 2, which produces a normalized off-axis deflection (i.e. ΔY/L in this case) of 0.493 in analytical solutions^41^, was applied. The SOCB approach, in which the mechanics solution is coupled to the curvature predictions, requires only 2 nodes to predict deflection with ~1% error. The jointed and FE approach, where linear approximations decouple the mechanics and curvature variables, require 50 and 200 nodes, respectively, to achieve similar errors. (**B**) Number of nodes required for achieving prescribed error levels at different % deflections (i.e. ΔY/L * 100) in the SOCB and jointed models: The SOCB model gives lower error levels even at lower node numbers, resulting in a higher physical accuracy with lower computational expense.

Consider a test case of a horizontal cantilever subjected to an off-axis point load P at its free end (Fig. 4A). The value of P is fixed such that PL^2^/EI = 2, a loading which produces a normalized deflection of 0.5 (e.g. ΔY/L = 0.5) in analytical models of large-strain cantilever bending^41^. For the prescribed cantilever loading, the SOCB model requires only 2 nodes to predict the –X and –Y deflections within 1% error of the analytical solution (Fig. 4A). By contrast the jointed approach, where the bending is restricted to occur at hinges between straight segments, requires as many as 50 nodes to get the error down to 6%. A Finite Elements (FE) method that solves large-deformation beam physics (Comsol Multiphysics software) needs 200 nodes and 100 nodes, respectively, to reach 6% error using 2^nd^ and 4^th^ order interpolation in displacement. The method requires 3D FE elements to handle large-deformation bending in 3D space, partly accounting for the high node numbers. It can be argued that the performance of the SOCB model (where a 6^th^ order interpolation in displacement is implied by Eq. 5) should be compared against FE approaches using 6^th^ order interpolation. However the SOCB approach achieves 6^th^ order interpolation using only two nodes per segment (i.e. with the node density of a linear FE element), whereas 6^th^ order interpolation in FE would require 6 nodes per element dimension and therefore a higher computational cost. It can also be argued that because the 1D linear type of beam elements in FE analysis use high-order interpolation function similar to Eq. 5 (but limited to the third power), and therefore should predict displacement and angle with reduced nodes. However, these FE elements are based on small-strain bending physics, the Hermite functions are used to interpolate displacement and not angles. Angles are instead approximated as differentials of displacement (see Eq. 3), thereby removing the proportionality between curvature and mechanics specified by large-strain Euler bending physics. While computationally cheap, the 1D beam elements do not converge to the large-deformation bending solution, and therefore were not included for comparing performances in Fig. 4A.

The predictions of the SOCB method for a coupled in-plane and out-plane beam loading problem is described in Supplementry Information 4, along with a plot of the error from numerical integration. Figure 4b compares the number of nodes required by the SOCB and jointed approach to reach a prescribed accuracy level for the entire gamut of % deflections (i.e. ΔY/L * 100). Since the end load is scaled  by cantilever length and bending stiffness, the number of nodes required to reach a desired error level is only dependent on the % deflection. The jointed model required as many as 20 nodes to achieve 10% error even at moderate deflections (<30%), whereas the SOCB model required only 2 nodes to achieve 1% error at those deflection levels. At higher deflections (>40%), the jointed model required ~30 nodes to achieve 10% error, but the SOCB model reached <1% error with these node numbers. The computation cost roughly scales as the second power of the degrees of freedom involved (i.e. ~(# nodes X # equations per node)^2^). Since about the same number of equations (mass, moment, and momentum conservation, and bending constitutive equation) is solved at each node, the number of nodes in Fig. 4A,B reflects the computation price paid for achieving physical accuracy in different approaches that solve large-deformation bending.

### Validating the generalization to arbitrary filament contours and in 3D

**Figure 5:**
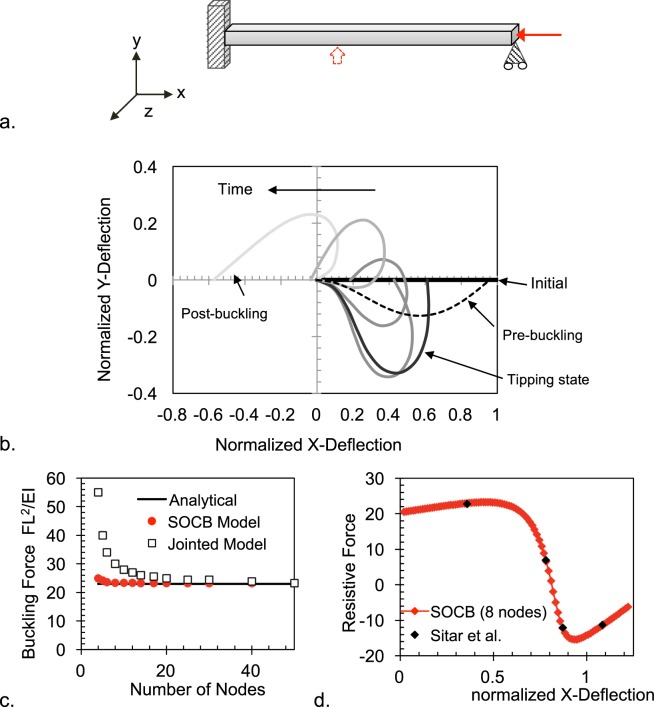
(**a**) Initial loading configuration of a unit cantilever for buckling deformation. In addition to the axial end-load that drives buckling, a small vertical force (dotted arrows) is applied at initial time steps to encourage slight curving of the cantilever under the axial load. (**b**) The bending configurations of the cantilever under axial compressive load are shown. When the axial load is less than the tipping force (shown for FL^2^/EI = 20 for the pre-buckling state shown in the figure), the cantilever resists with stable in-axis bending (shown in dotted lines). At the axial force of FL^2^/EI = 23.3, the cantilever reaches the tipping state, buckles and undergoes large deflections with large change in contours that stabilize at the post-buckled state (shown for 10 nodes). The X and Y deflections are normalized by the cantilever length. (**c**) The scaled axial force at which a unit cantilever is tipped into buckling is predicted for different node numbers using the SOCB and jointed approached. The tipping force is significantly over-predicted in jointed models at low node numbers. The analytical comparison is obtained from Sitar *et al*.^24^. (**d**) Resisting force vs. x-deflection produced as a unit cantilever buckles under a tipping load of FL^2^/EI = 23.3. The predictions of the SOCB model (shown for 8 nodes) match those reported by Sitar *et al*.^24^.

The accuracy of the SOCB approach to handle the moment arms in arbitrarily-curved segments can be validated by simulating the axial buckling of a filament. Consider the axially-loaded filament in Fig. 5a. It can resist compressive force with stable bending until a tipping configuration is reached. Once tipped, the filament buckles by passing through low-resistance states until the compressive force is resisted by filament tension in the opposite direction (Fig. 5b). During the course of buckling, the end forces change from compressive to tensile, segment angles shift quadrants, and moment arms change in magnitude and direction. Therefore axial buckling presents a good test case for validating the assignment of moment arms and force transformations in a method for solving large-strain bending. The SOCB approach predicts an evolution of segment contours during the course of buckling similar to those published elsewhere^24^ (Fig. 5b). The SOCB model is also able to predict the magnitude of the tipping force (scaled for filament length and EI) with <10% error using only four nodes, and with <1% error with eight nodes. The jointed approach, on the other hand, estimates the tipping force with >100% error when using four nodes, with the error stabilizing only beyond 40 nodes. The higher error from the jointed approach can arise from it systematically under-predicting bending deflections when a small number of nodes is used (Fig. 4). As a result, the force required to deflect the filament to the tipping point is over-predicted. Our results imply that since filament buckling is governed by tipping dynamics (i.e. large changes in deformation are produced when the force or deflection crosses a threshold), small errors in estimating the tipping force/deflection can produce disproportionally large differences in the perceived compliance of interconnected filamentous media and in the realistic prediction of their physics.

In the SOCB approach, we generalize the problem to 3D by decomposing all forces to the in-plane and out-plane components and determining the moment arms of each in the analysis frame. Now in spherical coordinates, the in-plane and out-plane bending directions are not mutually symmetric. However all our calculations for the in-plane and out-plane moments are symmetric or similar, except for when a cos term pre-multiplies the in-plane torques. We show that this correction is sufficient to capture the asymmetry between in-plane and out-plane bending. Consider the simple case of a cantilever loaded off-axis in the Y and X directions (Supplementry Information 4). The displacement solution will have to be calculated after converting the forces to spherical coordinates, solving contour angles in the two bending planes, and transforming back to rectangular coordinates. If any of these transformations or calculations were wrong, the output Y- deflection will not be the same as the output Z- deflection. We show in Supplementry Information 4, that the SOCB approach predicts similar Y- and Z- displacements for the Y- and Z- off-axis loading, even though the problem per se was solved by transforming to asymmetric spherical coordinates. While this validates how we pose and solve 3D bending in the SOCB approach, the accuracy and computational cost of the methods is also significantly improved compared to FE and jointed approaches (see Supplementry Information 4, where the deflection vs. node # profiles obtained for coupled in-plane and out-plane bending are very similar to those shown in Fig. 4A for the in-plane alone).

### Using SOCB approach to simulate mechanics of filamentous media such as the compression of dendritic actin networks

**Figure 6:**
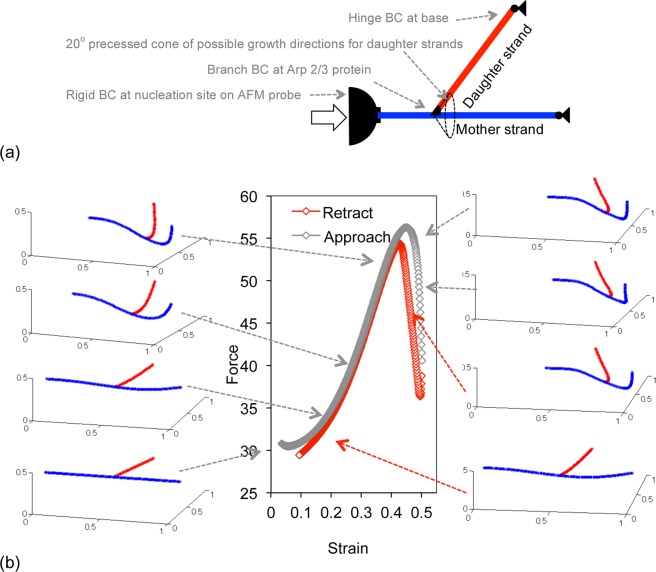
Compression of a unit actin mesh consisting of a single mother filament and daughter filament. The mother filament is stress and materially continuous, making the crosslink not a conventional pin joint. (**a**) Schematic of a unit dendritic mesh comprising of a mother filament outgrowing from a surface-adsorbed nucleation site on an AFM probe, and a daughter filament outgrowing from a cross-linker protein attached to the mother filament. The relative ϕ angle between the mother and daughter filament is 20^0^ at the site of crosslink. The filaments are assumed to stop growing when they hit a solid surface, and a hinge boundary condition is applied at the filament ends. The mother filament is 1 µm long with the daughter filament outgrowing every 0.5 µm distance to length of 0.5 µm. (**b**) The compression of a mother strand with a daughter appendage produced an initial region of low-force development followed by a nonlinear increase and a sharper downturn (contrast force curves in Figs 4D and 5b).

Figure 5d shows how the resisting force evolves when a single slender filament is axially compressed. The resisting force increases linearly with deflection as long as the bending is stable (<50% deflection in Fig. 5d), but makes a gradual downturn and decreases rapidly when the tipping force or contour is exceeded (>50% deflection in Fig. 5d). However, when a dendritic actin mesh outgrown from an AFM probe is compressed, the initial linear force increase (i.e. constant compressive stiffness) is followed by a nonlinear force increase and then a sharp downturn which is reversible^27^. The dendritic actin mesh consists of ‘mother’ strands that grow out from surface-adsorbed nucleation seeds, with ‘daughter’ strands off-shooting at 70° angle from existing strands (Fig. 6a). The reversible nature of the dendritic mesh collapse suggests that it arises from strands of actin buckling rather than from the mesh architecture changing on the time-scale of compression (e.g. breakage at crosslinks, actin treadmilling)^33,42–44^. The nonlinear stiffening of the compressing dendritic meshes has been attributed to the axial-stretching of actin strands between displaced crosslinks in the network^33,45–47^. However, to our knowledge, all three compression regimes of the dendritic actin mesh have not been reproduced in simulations.

For the purposes of describing the actin mesh in the SOCB method, the term ‘strand’ is used to refer to the physical actin fiber that may be contiguous through several branch points (e.g. the mother filament in Fig. 5a), as opposed to the term ‘filament’ which is a computational construct referring to the section of a slender element between branch points and free ends (see Supplementry Information 1).

Using the SOCB approach, we check if the simple addition of a ‘daughter’ strand as a branch appendage to a ‘mother’ strand could change the latter’s nonlinear bending so that the compressive force changes from linear and gradual collapse (as in Fig. 5d) to nonlinear and sharp collapse. Since the nodal variables include higher order differentials of angle, complex crosslink force-transfers can be prescribed with the SOCB method. Consider how force would transfer at a crosslink in a dendritic actin mesh. The crosslink is generated by an Arp2/3 protein binding to a pre-existing mother actin strand. The bound protein serves as the nucleation site for a daughter strand to grow off at 70° angle. In other words, the mother strand is materially continuous through the branch point and the daughter strand hangs off it like an appendage (Fig. 6a). The force transfer in such a crosslink can be captured by specifying that (a) the angles and moments at the ends of the two segments that are part of the mother strand match at the crosslink (i.e., mother strand is materially continuous through the crosslink and bends as one unit), (b) the net force at all three segment ends in the crosslink be balanced (i.e., the displacement of the crosslink is determined by the net force balance), and (c) the position and angle of the daughter strand is constant relative to the mother strand at the crosslink (i.e., the daughter strand hangs off at an 70° angle from the mother strand). The implementation of the branch boundary condition in the SOCB approach is described in Supplementry Information 5.

We test the compression behavior of the unit dendritic mesh shown in Fig. 6a, (i.e., an 0.5 µm daughter strand outgrown with ϕ = 20° from the middle of an 1 µm long mother strand) with the boundary conditions described. The compression of the mesh unit produced an initial region of low-force development followed by a nonlinear increase and a sharper downturn (compare force curves in Fig. 6b and Fig. 5d). The collapse in force reversed when the compression direction was reversed (see red curve in Fig. 5b and associated snapshots). Snapshots of the bending filaments at different points in the force curve verify that the mother filament remains materially continuous, i.e. the bending curve is continuous through the crosslink (Fig. 6b). In the region of nonlinear force increase, the daughter appendage appears to resist the continuous bending of the mother strand. The force downturn occurs when the daughter strand is no longer able to constrain the mother filament and the mother strand buckles. In other words, an axially-loaded element can be made nonlinear-stiffening with the mere inclusion of branch elements. However the unit dendritic mesh did not produce the linear force region that was observed in experiments to occur before the non-linear force increase.

**Figure 7:**
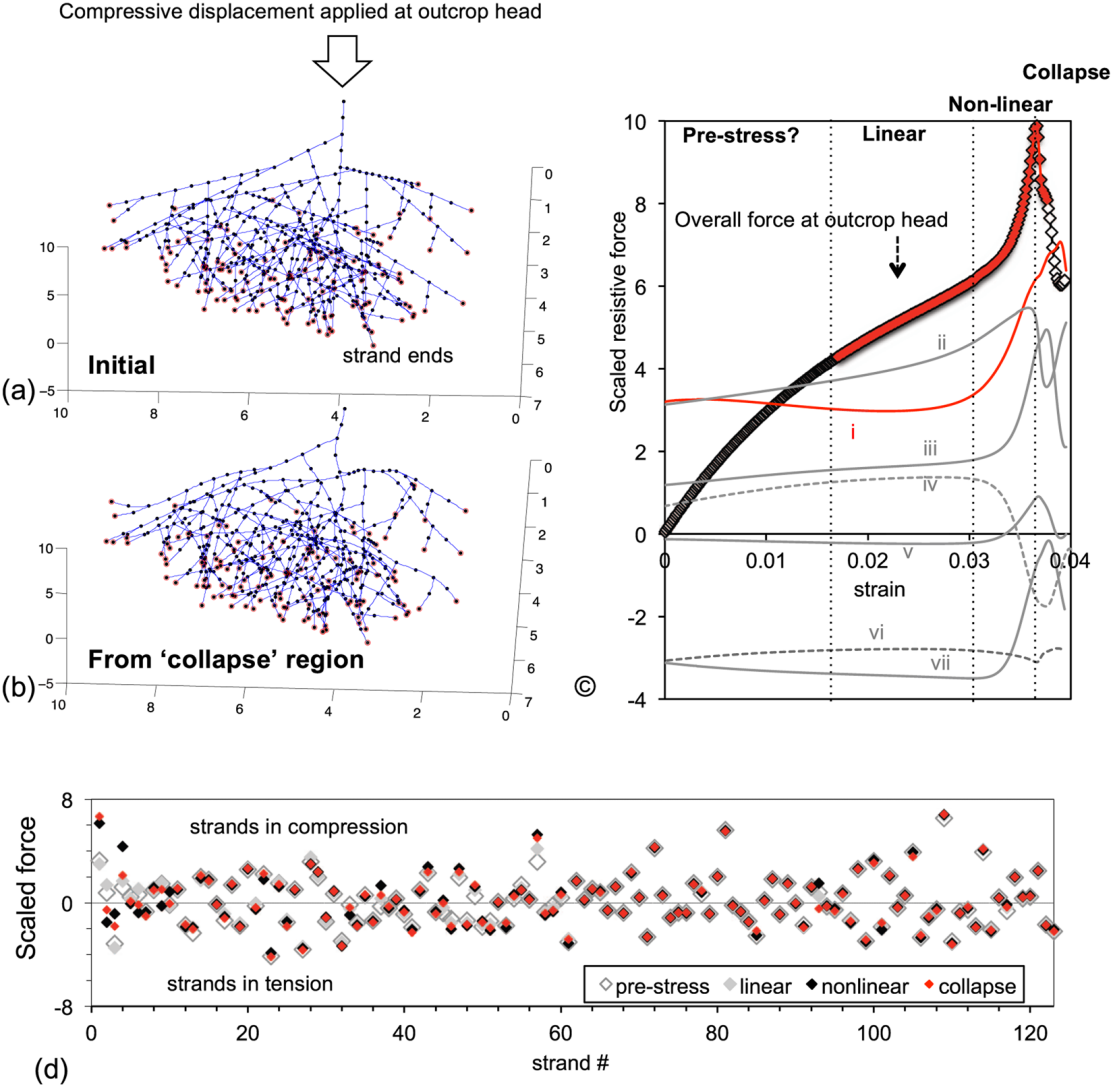
(**a**) An outcrop of dendritic actin mesh grown from one nucleation seed. The first-generation mother filament was grown at *θ* = 0. In every generation of growth, daughter filaments emerge from all existing filaments (i.e. mother filaments in that generation) at *ϕ* = 20° and arbitrary theta, and all filaments grow 1 µm. To restrict mesh size, daughter filaments growing towards the outcrop head were replaced. The outcrop was grown for eight generations, but with filaments halting when they reach 6 µm, to simulate the sample height used in experiments. Strand ends are encircled in red. The nodes are shown in black and a segment is 0.5 µm long. (**b**) A snapshot of the outcrop in the collapsed force region. (**c**) The overall force response determined at the outcrop head has four regions of which the latter three were observed in experiments (highlighted in red). Also shown alongside are the force contributions of individual actin strands sampled at the strand ends. The individual strands do not necessary follow the force response of the entire outcrop. The mother strand (red) exhibits a near flat force, without a ‘linear’ region, before rising nonlinearly (curve i). The mother filament collapse appears to occur after the overall outcrop does. Curve ii is from a daughter strand in compression which exhibits linear force increase before collapsing sharply. Curve iii is from a compressed daughter strand that exhibits all three force regimes. Curve iv is a compressed strand which collapses much before the outcrop does. Curve v is from a strand in tension and weakly load-bearing. Curve vi is a tensed strand that also collapses. Curve vii is a tensed strand that remains relatively unaffected even as the outcrop collapses. (**d**) A scatter plot of the forces at all strand ends is shown for four strains selected from the ‘pre-stress’, linear, nonlinear, and collapse region each. The x axis is a listing of all strand ends. The forces from several strands remain relatively unchanged (i.e. symbols marking force in different strains overlap) even as the overall outcrop is compressed, suggesting that few strands control the overall outcrop response.

We test how the compression of the unit dendritic mesh changes when it includes multiple generations of mother and daughter filaments. An outcrop of actin outgrowing from a nucleation seed adsorbed on an AFM probe, and into a 6 μm gap between the probe and opposing surface was simulated (Fig. 7a). Eight generations of dendritic actin were outgrown from a single nucleation seed, with the pre-existing mother filament ends and newly-branching daughter filaments growing 1 μm every generation. Strands were stopped growing when they exceeded the gap length of 6 μm. The resulting outcrop had continuous actin strands of varying length, from an 6-μm first-generation mother strand to an 1-μm eighth-generation daughter strand. A small lateral force, which was significantly smaller than Brownian forces, was applied on all strands in the in-plane and out-plane directions to induce them to curve when compressed. The 6 μm outcrop was compressed at a steady rate of 100 nm/sec (Fig. 7b), and the force detected at the outcrop head was profiled (black diamond symbol in Fig. 7c). Following an initial region where the force rise is convex, the three force regions that were observed in experiments were reproduced (highlighted with red diamonds in Fig. 7c). The initial region of convex force increase (‘pre-stress’), where straight strands in simulation are compressed into curved shapes in simulation, could be missing in experiments because strands start out already curved there from Brownian forces.

The force response of individual strands, measured at their ends on the lower boundary of the gap (shown in red in Fig. 7a), do not necessarily trace the overall response measured at the outcrop head. For instance, the force curve of the original mother strand is initially flat and not concave or linearly increasing. The eventual non-linear stiffening of the mother filament is followed by collapse, but at compressions higher than when the outcrop collapses (red curve in Fig. 7c). In fact, the overall outcrop response seems to be a cumulative effect of the force response from individual strands, which may be in tension or compression, may or may not have a nonlinear force rise, and may or may not collapse (grey curves in Fig. 7c). In other words, the outcrop ‘collapse’ is not a failure of the entire mesh, but a redistribution of forces that occurs with few strands buckling. Consistent with this idea, the scatter plot in Fig. 7d shows that the forces on several strand ends remain virtually unchanged (i.e. symbols overlap) even as the overall outcrop goes through different force regimes. In other words, since not all strands equally determine the outcrop response, the force transmission in a dendritic mesh can be rapidly rerouted (e.g. fluidized) by cleaving only select ‘load-bearing’ strands. By corollary, large changes in the overall mesh mechanics (e.g. cell stiffness) can be induced without changing the tension/compression of all strands.

We note that the magnitude of force response in experiments maybe larger than that in the above simulation because: (i) the force-response in experiments is contributed by multiple outcrops which may be physically entangled into each other, (ii) the distance between branching may be smaller and more randomized in experiments, and (iii) the compression in experiments was applied by oscillating at constant frequency and increasing the target stresses, as a result of which the strain-rate and therefore the hydrodynamic drag would increase at higher stresses. Moreover it has been reported that branching in a dendritic actin mesh is biased to the concave side of curved mother strands^48^. We did not include such biasing in our network generation, and the effect of such biasing on the network mechanics is not clear. Future versions of the model will include the effect of Brownian forces.

## Discussion

There has been pioneering advances in the modeling of biological networks^15,18,34,49–52^. While many of these works focused on the nonlinear behavior of fibrous *networks*, our focus was to capture the nonlinear bending of *filaments* within the networks with high fidelity. In other words, if we cut out any filament segment from a network model and simulate its compression, would it give the same geometry (i.e. shape) and mechanics (resistance) of a classic buckling beam? In many nonlinear network models, the bending mechanics of a constituent filament is approximated. The filament can be idealized as an axial spring with bilinear elasticity (i.e., lower elasticity in compression than tension); in such case the filament resistance increases linearly with axial compression^18,49,53,54^. But according to Fig. 5d, the resistance of a buckling filament falls precipitously at ~50% deflection, and then changes direction to favor buckling. Moreover, the axial spring idealization does not allow out-of-axial bending and force transfers. The jointed rod is another popular idealization used for filaments in cellular and matrix networks^15,31,55^. Here the bending filament is treated as two straight rods hinged at a joint. While it captures out-axis bending, the idealization is equivalent to a jointed model with 3 nodes in Fig. 4a, which was found to under-predict transverse deflection by ~50%. Also since the SOCB approach retains higher-order angle differentials as primary variables, one could go beyond the conventional pin-joint crosslink to simulate more realistic force transfer such as that occurring in dendritic actin architecture. We note that the 2D version of this continuous beam approach was proposed by Chandran and Mofrad^56,57^, also under the premise that any isolated section of the filament should be able to simulate Euler bending and rod diffusion physics with high fidelity. This approach was named as Rods on String idealization. However the force and moment assignment in the 2D version had to be vastly reconfigured in the SOCB model to capture the coupled out-plane and in-plane bending physics in 3D settings. Finally, while the SOCB approach also uses interpolation polynomials like in FE approaches, these are not shape or basis functions which reach maximal value at a FE node and which multiply the conservation equations to distribute and reduce the latter’s differentiation order.

The central idea of the SOCB approach is to achieve high order continuity in angle by linking the variables that are already solved in moment and force balance equations, instead of adding more nodes like in FE. As a result, the number of nodes required to solve nonlinear bending is decreased significantly. Results show that when an insufficient number of nodes is used to capture large-strain bending, the X-deflection get under-predicted and the length of the bending element increases artifactually. Also, the Y-deflection gets under-predicted and the bending element appears stiffer. These observations imply that when modeling large filamentous assemblies, underestimating the nonlinear bending of filaments can increase the microstructural resistance to rearranging under internal forces (for instance in the case of myosin remodeling of cellular networks) or decrease the compliance of soft matrices and civil-engineering structures to external loading^58–62^.

The compression of an actin outcrop provided a relevant case study to evaluate the proposed approach. It is a problem involving many arbitrarily-arranged filaments, connected by crosslinks which are not traditional pin-joints, with a macro-scale nonlinear force collapse resulting from large-strain micro-scale bending, and will all the macro-scale force features not captured in simulations hitherto. Simulations of a unit dendritic actin mesh shows that adding a daughter filament as an appendage to a buckling mother filament restricts the nonlinear bending of the latter, causing the compressive force to increase nonlinearly. Eventual buckling of the mother strand causes the force fall, but in a reversible manner. An outcrop of dendritic actin with eight generations of growth and branching, i.e. a total length of ~150 μm, can be simulated with the computing power of a regular laptop. The linear and nonlinear increase in force and collapse detected at the outcrop head appears to be a cumulative effect of different load-bearing profiles in individual strands, with loads redistributing as individual strands buckle. Interesting the compression force profile was mostly determined by a few fibers, and the load in majority of the strands remained unchanged. This result is reminiscent of findings regarding the nature of tensile force-transfers between cells in fibrous matrices, that it is mostly carried by few fibers, which in this case are brought to align with the direction of tension^49,63^.

## Supplementary information

Supplementary Information
