# Supplementary material for: Capturing 3D large-strain Euler-bending filament dynamics in fibrous media simulations; sample case of compression collapse in dendritic actin network
Source: Sci Rep. 2019 Mar 8;9:3990. doi: 10.1038/s41598-019-40430-y (PMC6408500; doi:10.1038/s41598-019-40430-y)
Supplement: Supplementary file 1 — Supplementary Information [file 41598_2019_40430_MOESM1_ESM.pdf]

SUPPLEMENTARY INFORMATION

Capturing 3D large-strain Euler-bending filament dynamics in fibrous media simulations; sample case of compression collapse in dendritic actin network

Jyothirmai J. Simhadri, Preethi L. Chandran*

[1] *Department of Chemical Engineering, Howard University, Washington, DC 2005*

Corresponding author

Preethi L. Chandran, PhD

Assistant Professor
Department of Chemical Engineering, College of Engineering and Architecture, Howard University
Department of Biochemistry and Molecular Engineering, College of Medicine, Howard University

Address:
1011 LK Downing Hall
2300 6th Street, NW, Howard University
Washington, DC 20059
Email: preethi.chandran@howard.edu
Phone: 202-806-4595

SUPPLEMENTARY INFORMATION 1

TASK FLOW IN THE SOCB APPROACH:

The flow of tasks for solving filament mechanics using the SOCB method is shown below:

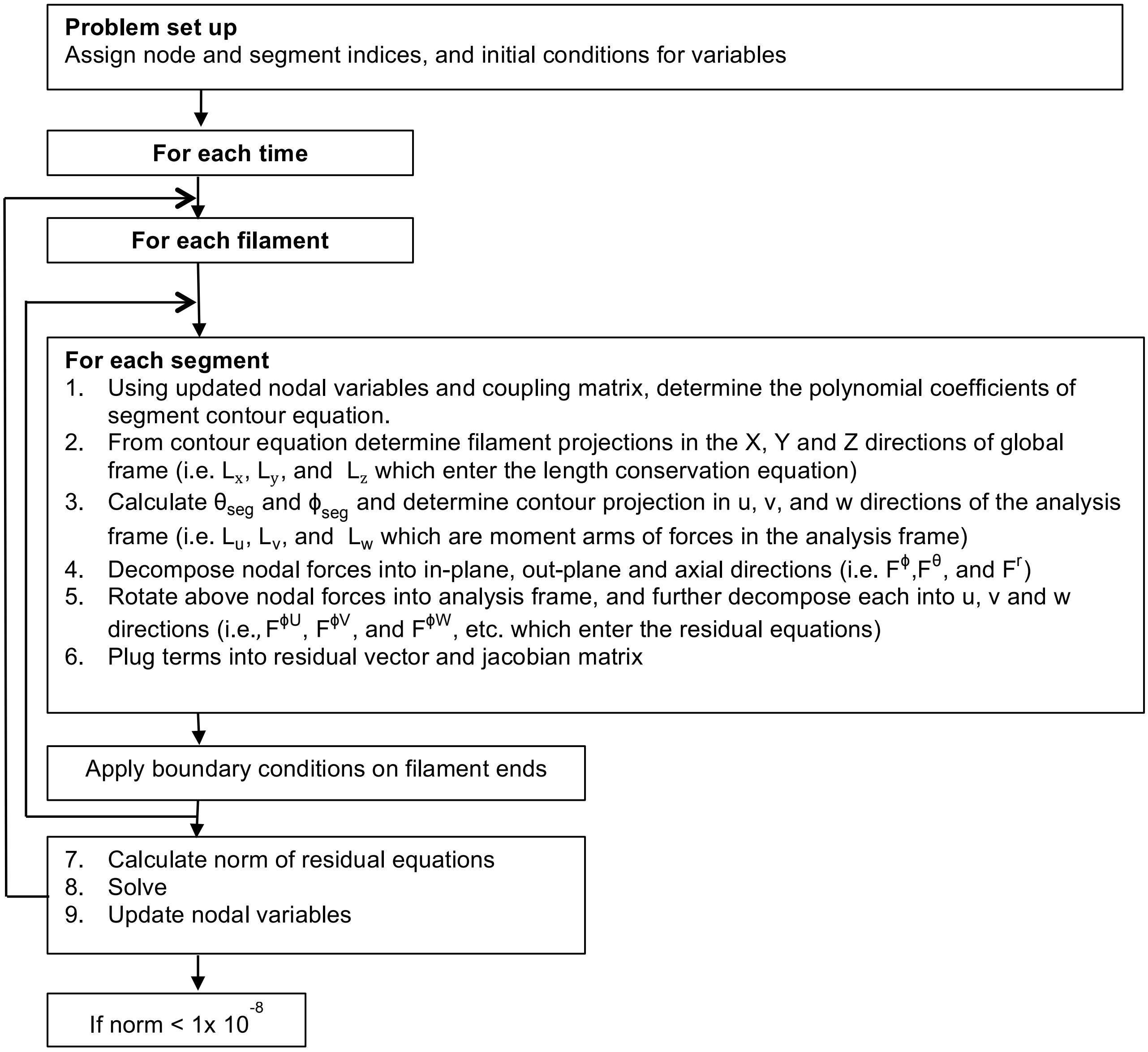

Suppl. Fig. 1a Flow chart of tasks in the SOCB approach.

Each task in the flow chart is described in detail below.

Step 1: Problem setup:

Each stretch of slender element between crosslinks or free ends is considered a filament (Suppl. Fig. 1b). A filament can be further sectioned into segments over which the contour is determined to fifth order continuity. The conservation (length, force, moment) and constitutive equations are solved for each segment (Suppl. Fig. 1b). The ideal number of segments is

determined by two constraints: [i] for the accurate calculation of moment arms there should be only one inflexion per segment, [ii] the maximum geometrical accuracy achievable per segment is 5th order continuity in θ and ϕ separately. Each segment i is flanked by nodes i and i+1 at which variables are solved. The table below lists the equations assembled at each segment, the principle variables of beam mechanics being solved at each node, and the counterpart variables 'coupled' to filament conformation.

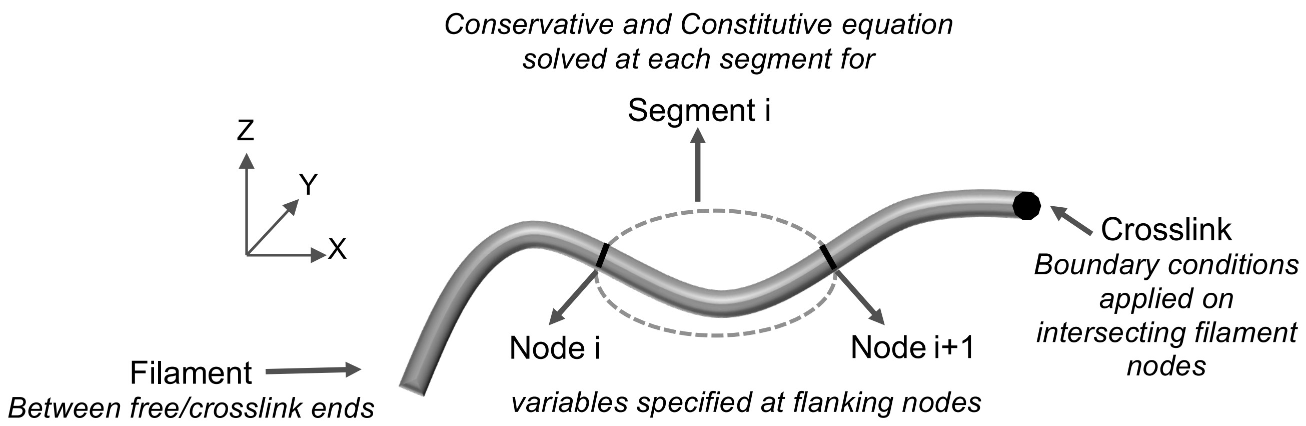

Equations	Variables solved at node i	Coupling to filament contour at node i
Length conservation	Projection along x – axis: X_i<br>Projection along y – axis: Y_i<br>Projection along z – axis: Z_i	
Bending Constitutive equation	In-plane: θ_i<br>Out-plane: Φ_i	
Moment balance	In-plane: M_i^{θ}<br>Out-plane: M_i^{ϕ}	$= -\frac{d\theta}{ds_i}$<br>$= -\frac{d\phi}{ds_i}$
Force Balance	Along u – axis: $F^{\phi U}, F^{\theta U}, F^{rU}$<br>Along v – axis: $F^{\phi V}, F^{\theta V}, F^{rV}$<br>Along w –axis: $F^{\phi W}, F^{\theta W}, F^{rW}$	$= -\frac{d^2\theta}{ds^2_i}$<br>$= -\frac{d^2\phi}{ds^2_i}$

Suppl. Fig. 1b Problem setup for the SOCB approach: [top] A continuous filament is sectioned into segments with mechanics solved over each segment and variables determined at each node. Boundary conditions are applied at filament ends that are free or part of crosslinks. The schematic for assigning node and segment indices is illustrated. [bottom] The table lists equations solved for each segment. The equations determine nodal variables describing beam mechanics. The mechanics variables are proportional to variables describing the nonlinear filament contour. Forces and moments are scaled by EI.

Step 2: Transformation of forces and moments

The forces and moments at a node are transformed from global X-Y-Z coordinates to the radial, in-plane and out-plane directions at the node (illustrated in Fig. 3a of the main text). The radial direction at a node is tangential to the filament at the node. The in-plane direction is normal to the filament and aligned along the latitude of a spherical surface passing through the node. The out-plane direction is normal to the filament, but aligned along the longitude of the spherical surface through the node. By decomposing the forces and rotations into in-plane and out-plane, the moment balances and bending constitutive equations can be separately written for the two bending directions.

Step 3: Backing out the filament contour from variables of beam mechanics solved at each node in previous iteration

Solving the constitutive, moment-balance, and force-balance equations over a segment i makes available the variables $\theta[s]$, $\phi[s]$, $d\theta/ds$, $d\phi/ds$, $d^2\theta/ds^2$, $d^2\phi/ds^2$ at both nodes i and i+1 of the segment (Suppl. Fig. 1b). 's' is the variable tracking distance along the segment contour. Therefore, in a segment there are six variables each for the in-plane and out-plane angles. The six variables each can be used to fit the coefficients of two fifth-order polynomials that would describe how $\theta[s]$ and $\phi[s]$ changes within a segment (Eq.1).

$$\begin{aligned}\theta(s) &= a^{\theta} + b^{\theta}s^{1} + c^{\theta}s^{2} + d^{\theta}s^{3} + e^{\theta}s^{4} + f^{\theta}s^{5} \\ \phi(s) &= a^{\phi} + b^{\phi}s^{1} + c^{\phi}s^{2} + d^{\phi}s^{3} + e^{\phi}s^{4} + f^{\phi}s^{5}\end{aligned} \tag{1}$$

The coefficients are solved by substituting s = 0 for values at node i, and s = l for values at node i+1 in Eq.1 and its differentiated versions. Here l is the length of a segment. The vector-matrix equation to solve the coefficient of $\theta[s]$ would look like:

$$\begin{bmatrix} \theta_i \\ M_i^{\theta} \\ V_i^{\theta} \\ \theta_{i+1} \\ M_{i+1} \\ V_{i+1}^{\theta} \end{bmatrix} = \begin{bmatrix} \theta_i \\ \frac{d\theta}{ds}_i \\ \frac{d^2\theta}{ds^2}_i \\ \theta_{i+1} \\ \frac{d\theta}{ds}_{i+1} \\ \frac{d^2\theta}{ds^2}_{i+1} \end{bmatrix} = \begin{bmatrix} 1 & 0 & 0 & 0 & 0 & 0 \\ 0 & 1 & 0 & 0 & 0 & 0 \\ 0 & 0 & 1 & 0 & 0 & 0 \\ 1 & l & l^2 & l^3 & l^4 & l^5 \\ 0 & 1 & 2l & 3l^2 & 4l^3 & 5l^4 \\ 0 & 0 & 2 & 6l & 12l^2 & 20l^3 \end{bmatrix} \begin{bmatrix} a^{\theta} \\ b^{\theta} \\ c^{\theta} \\ d^{\theta} \\ e^{\theta} \\ f^{\theta} \end{bmatrix} \tag{2}$$

The matrix is essentially the ‘coupling matrix’ that couples the mechanics variables to the filament contour variables. A similar vector-matrix equation couples the out-plane mechanics to the out-plane contour variables. From the θ[s] and ϕ[s] functions, the X- Y- Z- displacement variables are obtained using standard spherical coordinate projections as part of the mass-conservation equations below (Eq. 3).

Step 4: Calculating contour projections in the global frame

For an inextensible filament, the mass conservation equations simplify to segment length conservation equations, since the filament diameter is essentially unaffected.

SOCB length conservation equations:

$$\begin{aligned} X_{i+1} - X_i &= \int_0^l \cos\theta(s)\cos\phi(s)\,ds \\ Y_{i+1} - Y_i &= \int_0^l \sin\theta(s)\cos\phi(s)\,ds \\ Z_{i+1} - Z_i &= \int_0^l \sin\phi(s)\,ds \end{aligned} \tag{3}$$

The expression for θ[s] and ϕ[s] are given in Eq. 3. Since the integration is over the segment length l, the filament inextensibility is intrinsically maintained. The integration is performed numerically for pre-specified integration intervals.

Step 5: Calculating contour projections and force components in the analysis frame

To generalize the calculation of the moment arms when the segment is arbitrarily-curved in 3D space, the segments are rotated into an analysis frame. The segments are rotated through the ϕ and the θ of the line joining their two ends, so that the end nodes line up along the u direction of the u, v, w coordinate frame. This rotation by ϕ_{seg} and θ_{seg} ensures that the in-plane and out-plane character of the forces are conserved after the rotation. This is because a -ϕ rotation occurs along the longitude of a sphere (thereby preserving the longitudinal direction of the out-plane forces), while the direction of the turning axis is the same as the in-plane force (thereby leaving the latitudinal direction of the in-plane force unchanged during the rotation). In the absence of torsional rotation, the in-plane direction is always the cross-filament direction that lies in the global X-Y plane or analysis u-v plane. The rotated in-plane, out-plane, and axial forces are each further decomposed into u, v, and w components in the analysis frame. In this frame, the shadows of the segment in the u, v, and w direction directly provide the moment arms for the u, v, and w components of the forces.

$$
\begin{aligned}
L_U^i &= \int_0^1 \cos\left[\theta(s) - \theta_{seg}^i\right]\cos\left[\phi(s) - \phi_{seg}^i\right]ds \\
L_V^i &= \int_0^1 \sin\left[\theta(s) - \theta_{seg}^i\right]\cos\left[\phi(s) - \phi_{seg}^i\right]ds \\
L_W^i &= \int_0^1 \sin\left[\phi(s) - \phi_{seg}^i\right]ds
\end{aligned}
\qquad (4)
$$

Step 6: Set up of conservation equations and constitutive equation in analysis frame
Fig. 3(d) (from the main paper) illustrates moment arms for each force component assigned from the segment projections along the u, v, and w directions; i.e. Lu, Lv, and Lw. We note that the moment arms for the in-plane force have to be in the in-plane or u-v plane. Similarly, the moment arms for the out-plane has to be in the u-w plane. An axial or tangential force at a node can bend a segment if it has an inflexion, i.e. a non-zero Lv and/or Lw. The moment arm of each force component and the moment equation it contributes to are tabulated in Table 1 in the main text. The force and moment conservation equations are obtained as a static balance of these components (Eqs. 10 and 9 of the main document). Typically simulations that idealize sections of continuous filaments as straight rods require a constitutive equation to relate local bending moment to the angle change. However in the SOCB method, where the filament remains continuous, the local angle is trivially the integral of the local curvature or moment. The angle-change or bending constitutive equation is written as the integral of the moment conservation equations (Eq. 8 in main document).

Step 7: Solving to update nodal variables and iterating
Boundary condition on the nodal variables are applied at the filament ends which are the free ends or part of crosslink. A slender element that is continuous through a crosslink is still treated as two filaments with their respective nodes overlapping, and the boundary conditions are applied to capture the material continuity. The equations are solved simultaneously by Newton Raphson iteration with manually-derived differential equations for the Jacobian.

SUPPLEMENTARY INFORMATION 2

REDUCTION OF SOCB APPROACH TO JOINTED MODELING

It is instructive to see when the SOCB formulation reduces to the jointed model case. In a segment of SOCB approach the filament angle does not vary at segment intersections but varies within a segment. In a section in the jointed approach, the filament angle varies between sections and not within a section. Therefore, the filament depiction in the SOCB approach morphs to that in the jointed approach when each rigid section is treated as one filament with one segment, and a hinge boundary condition is applied between filaments (Suppl. Fig. 2).

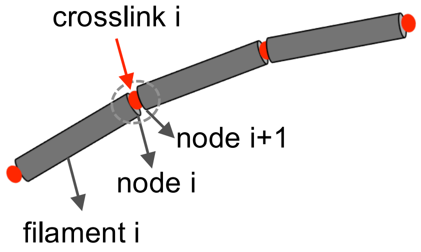

Suppl Fig. 2 Reduced depiction of the SOCB filament idealization for solving bending problems with the jointed filament approach.

In the jointed filament approach, the segment is a unbending rod and the polynomial describing θ[s] and ϕ(s) within a segment reduces to:

$$\theta(s) = a^{\theta}$$
$$\phi(s) = a^{\phi} \tag{5}$$

In other words, there are no coefficients in the filament curvature equation for the solution of the mechanics problem to feedback into. The mechanics-curvature coupling equation (Eq. 6 in main document) reduces to:

$$\begin{bmatrix} \theta_i \\ M_i \\ V_i^{\theta} \\ \theta_{i+1} \\ M_{i+1} \\ V_{i+1}^{\theta} \end{bmatrix} = \begin{bmatrix} \theta_i \\ \frac{d\theta_i}{ds} \\ \frac{d^2\theta_i}{ds^2} \\ \theta_{i+1} \\ \frac{d\theta_{i+1}}{ds} \\ \frac{d^2\theta_{i+1}}{ds^2} \end{bmatrix} = \begin{bmatrix} 1 & 0 & 0 & 0 & 0 & 0 \\ 0 & 0 & 0 & 0 & 0 & 0 \\ 0 & 0 & 0 & 0 & 0 & 0 \\ 0 & 0 & 0 & 0 & 0 & 0 \\ 0 & 0 & 0 & 0 & 0 & 0 \\ 0 & 0 & 0 & 0 & 0 & 0 \end{bmatrix} \begin{bmatrix} a^{\theta} \\ b^{\theta} \\ c^{\theta} \\ d^{\theta} \\ e^{\theta} \\ f^{\theta} \end{bmatrix} \tag{6}$$

The static equations of force and moment balance (Eq. 10 and 8 in main text) are equally applicable for the jointed case, however with the force and moment terms due to off-axis projections L_w and L_v becoming zero for a straight section. Instead of the bending moment being the differential of angle at any point on a segment (Eq. 9 in main text), it becomes the difference in the angle at the hinges for the jointed model case:

$$M_i^{\theta} = \frac{\theta_{i+1} - \theta_i}{\Delta l}$$
$$M_i^{\phi} = \frac{\phi_{i+1} - \phi_i}{\Delta l} \tag{7}$$

SUPPLEMENTARY INFORMATION 3

NEED FOR COMPRESSING THE IN-PLANE MOMENT ARM AFTER ROTATING INTO THE ANALYSIS FRAME

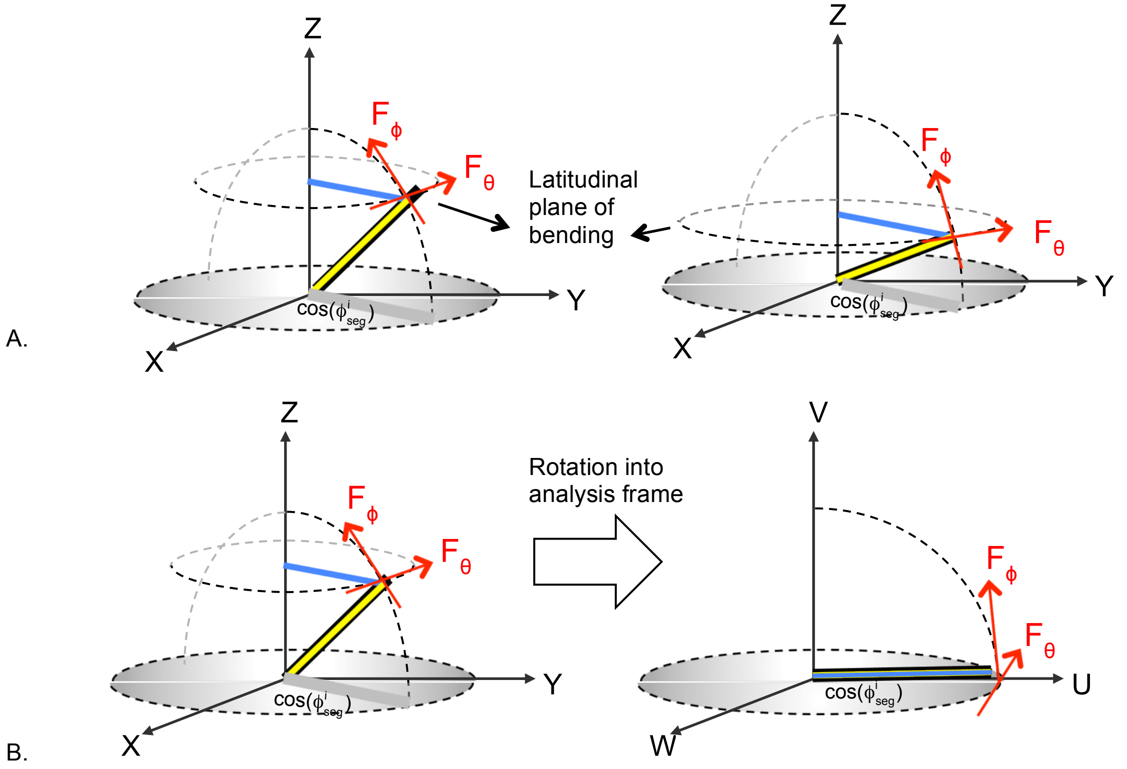

Suppl. Fig. 3 | [A] Schematic showing that the moment arm of the in-plane force (blue line) for bending in the latitudinal plane increases as $\cos\left(\phi^i_{seg}\right)$, where ϕ^i_{seg} is the angle made by segment i with respect to the X-Y plane. The principle is depicted using a straight rod as the segment (in black). The moment arm for the out-plane force (yellow) is the rod itself and it remains unchanged through the rotation. [B] When a filament is rotated by ϕ^i_{seg} into the u-v-w analysis frame, the moment arm for the in-plane force becomes longer in the analysis frame. Multiplying all in-plane moment arms by $\cos\left(\phi^i_{seg}\right)$ restores the arm lengths to that in the original X-Y-Z frame.

SUPPLEMENTARY INFORMATION 4:

VALIDATION OF IN-PLANE AND OUT-PLANE MOMENT CALCULATION

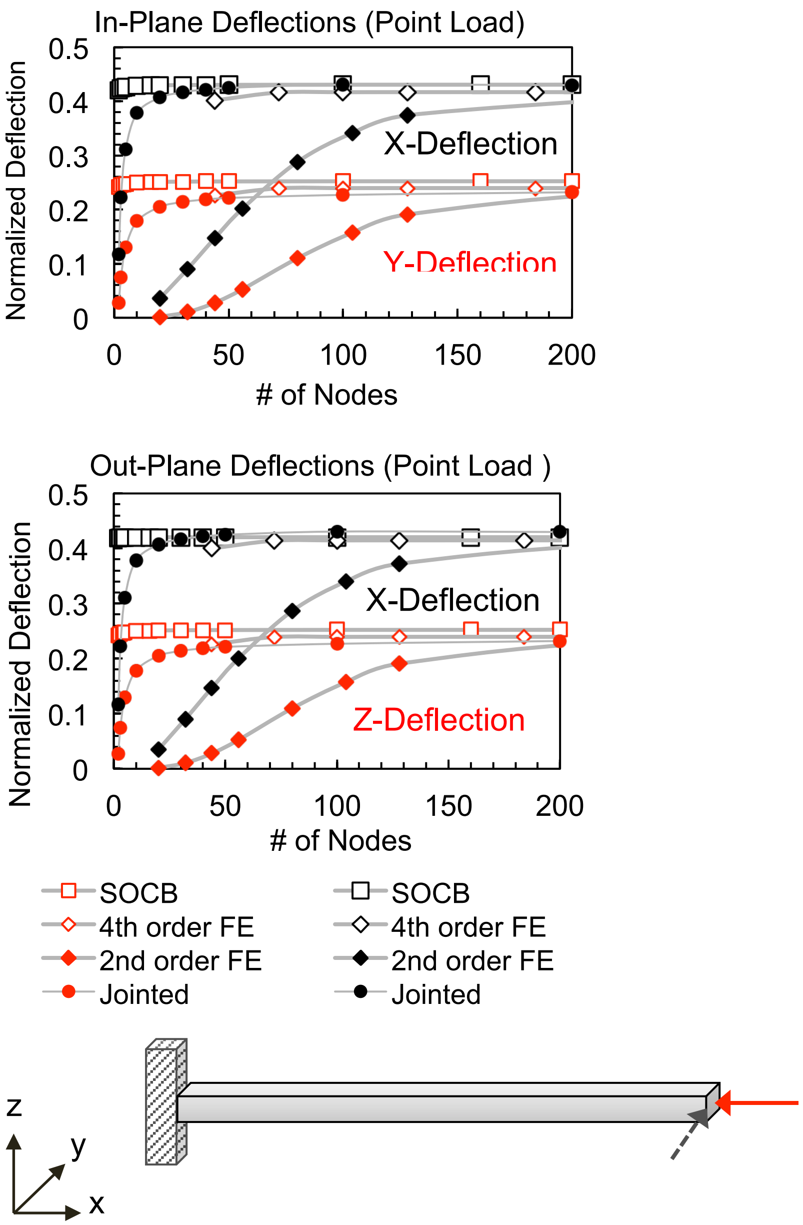

Suppl. Fig. 4A Predicting the z and y deflection of a horizontal cantilever that is separately loaded to the same extent in the z and y directions. In the SOCB approach, the z and y forces are transformed into out-plane and in-plane forces to solve the filament contour in those two planes (Eq.1). Even though the calculations in the in-plane and out-plane directions are not symmetric in spherical coordinates, the final displacements predicted for z and y directions are equal, consist with the application of equal forces in both directions. The ability to predict a symmetric solution using an asymmetric calculation-space validates the back and forth transformations between rectangular and

spherical coordinate systems in the SOCB approach. The SOCB approach predicts deflections with significantly less error using significantly less nodes than conventional approaches.

ERROR WITH NUMERICAL INTEGRATION OF DISPLACEMENT

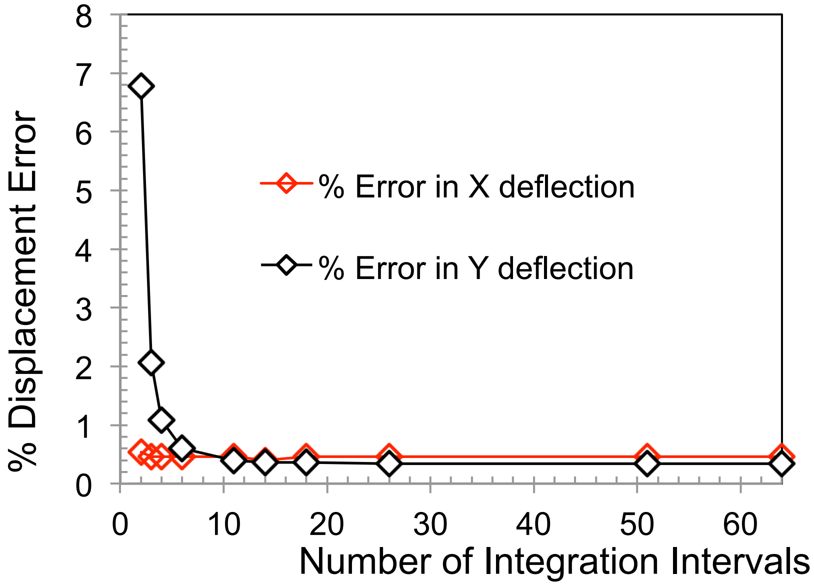

Suppl. Fig. 4B plots the error in cantilever deflections as a function of the number of integration intervals used for numerically integrating displacements in the length conservation equation (Eq. 7 in main text). A 1μm cantilever is subject to an end load that produces 50% deflection in analytical models. The cantilever was resolved as two segments. The y axis give the overall error in the simulated x and y deflection of the cantilever as the number of integrating intervals is systematically increased in each of its 0.5μm segment. Integration is performed using the center average of each interval. The error stabilizes to less than 0.5% error around 10 integration intervals; i.e., at an integration step of 0.05 μm for a 0.5μm segment (i.e.,) in a 1μm bending filament.

SUPPLEMENTARY INFORMATION 5

ASSIGNING BOUNDARY CONDITIONS AT CROSSNODES IN SOCB APPROACH

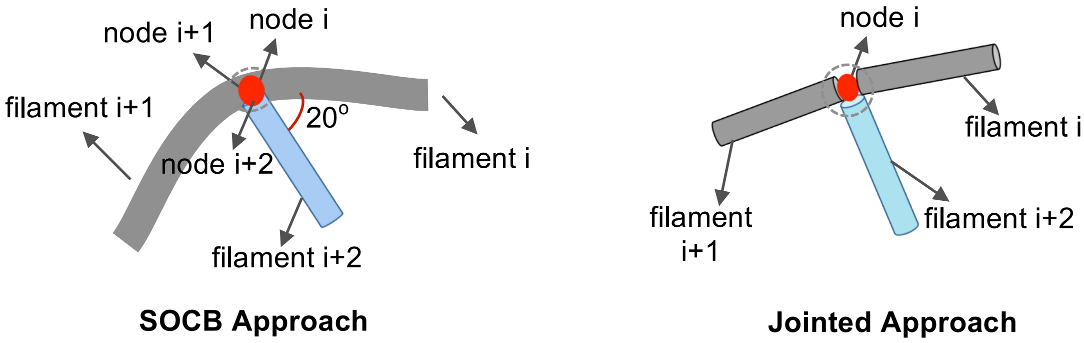

Suppl. Fig. 5 Configuration of crossnode in the SOCB scheme and in conventional approaches such as the jointed.

Figure 5 shows the differences between the way crosslinks are configured in the SOCB (left) and conventional approaches such as the jointed (right). In jointed approaches, there are three separate filaments that meet at one crossnode. The force and moment balance equations between all three filaments are solved at the one node for each crosslink (Suppl. Table 1). The crossnode therefore is an archetypal pin-joint joining three separate filaments. In the SOCB approach, a crossnode is the superposition of the end nodes of three filaments. This is because in the SOCB method, each node carries information of the angle, curvature, etc. of a filament end. Boundary conditions need to be applied to describe how each of the end nodes at the crosslink are related to each other, and therefore how the filaments intersecting at the crossnode are joined to each other. Therefore, if f is the number of filaments intersecting at a crossnode, there are f X 5 BCs prescribed for each crosslink in the SOCB approach, whereas only 1X 5 BCs can be specified in the jointed approach. The SOCB approach therefore affords large flexibility for assigning how the filaments at a crosslink are related to each other. In the case of a dendritic actin mesh, the crossnode is of the branch type with two filaments constituting a continuous mother filament (Suppl. Fig. 5 left). Therefore the boundary condition on the main node i from the mother filament specifies that the curvatures or moments of nodes i and i+1 (where i+1 is the daughter filament's end node at the crosslink) matches to capture the stress continuity between the strands. The boundary conditions on the other nodes denote how their position and angles match with the main node (Suppl. Table 1).

**SOCB**	**Jointed**
on node i from main segment of mother strand<br>$\frac{d\theta}{ds}_i = \frac{d\theta}{ds}_{i+1}; \frac{d\phi}{ds}_i = \frac{d\phi}{ds}_{i+1}$<br>$\sum_{n=i}^{i+1} F_n^u = 0; \sum_{n=i}^{i+1} F_n^v = 0; \sum_{n=i}^{i+1} F_n^w = 0$<br><br>on node i+1 from second segment of mother filament:<br>$x_i = x_{i+1}$<br>$y_i = y_{i+1}$<br>$z_i = z_{i+1}$<br>$\theta_i = \theta_{i+1}$<br>$\phi_i = \phi_{i+1}$<br><br>on node i+2 from daughter filament:<br>$x_i = x_{i+2}$<br>$y_i = y_{i+2}$<br>$z_i = z_{i+2}$<br>$\theta_{i+2} - \theta_i = \theta_{i+2}^{initial} - \theta_i^{initial}$<br>$\phi_{i+2} - \phi_i = \phi_{i+2}^{initial} - \phi_i^{initial}$	on node i intersecting all three filaments f at crossnode<br><br>$\sum_{f=i}^{i+2} M_f^\theta = 0; \sum_{f=i}^{i+2} M_f^\phi = 0$<br>$\sum_{f=i}^{i+1} F_f^u = 0; \sum_{f=i}^{i+1} F_f^v = 0; \sum_{f=i}^{i+1} F_f^w = 0$

Suppl. Table 1: Differences in how the BCs are specified on a crosslink in the SOCB (shown for branch crosslink) and jointed approach.
